# Decoding the anti-thrombotic effects of leonurine: a multimodal approach combining TCM repositioning and mTOR signaling

**DOI:** 10.1186/s13020-025-01160-8

**Published:** 2025-07-01

**Authors:** Xin Dong, Shuaibing Jia, Leifeng Zhang, Yong Liang, Jianhua Zhang, Yi Zhun Zhu

**Affiliations:** 1 Faculty of Innovation Engineering, Macau University of Science and Technology, Avenida Wai Long, Taipa, Macau, 999078 China; 2 School of Pharmacy, Macau University of Science and Technology, Avenida Wai Long, Taipa, Macau, 999078 China; 3 Medical Engineering Technology and Data Mining Institute, Zhengzhou University, 100 Science Avenue, Zhengzhou, 450000 China; 4 Peng Cheng Laboratory, Shenzhen, 518055 China

**Keywords:** Heterogeneous network, Traditional Chinese medicine (TCM), Repositioning, Ingredients-diseases relationship, Leonurine, Thrombosis, mTOR

## Abstract

**Background:**

As a natural small molecule compound, Leonurine have great potential for application in the treatment of cardiovascular diseases. However, there is still a gap in the treatment of thrombosis with Leonurine.

**Methods:**

A multimodal heterogeneous network is constructed using ETCM and STRING databases, integrating herbs, ingredients, targets and diseases. A reposition model constructed by random walk and random forests is used to predict the relationship between Leonurine and diseases. In addition, network pharmacology, molecular docking and molecular dynamics are used as computer-aided methods to confirm the target of Leonurine. Finally, CCK-8 assay, Flow cytometry, Western Blotting, and mouse experiments are used to validate the therapeutic potential of Leonurine from the perspective of biological wet experiments.

**Results:**

Firstly, based on the accurate prediction results evaluated by indicators, Leonurine is evaluated to have potential therapeutic effects on thrombotic diseases. Through computer-aided methods, mTOR is identified as a potential regulatory factor and may have a similar regulatory mechanism to the marketed drug Everolimus. Experimental results demonstrate that Leonurine reduces thrombotic cell apoptosis and promotes endothelial cell proliferation by inhibiting mTOR signaling. Additionally, in vivo studies show decrease mTOR expression in thrombotic tissues following Leonurine treatment.

**Conclusions:**

These findings underscore mTOR’s critical role in mediating Leonurine’s anti-thrombotic effects, supported by both computational and experimental evidence. The study provides a foundation for the application of TCM-derived compounds in modern medicine, particularly in thrombosis treatment.

**Supplementary Information:**

The online version contains supplementary material available at 10.1186/s13020-025-01160-8.

## Introduction

Thrombosis is a common symptom of cardiovascular disease and can lead to myocardial infarction, venous thromboembolism and other diseases [1]. They are a significant contributor to the global burden of disease [2]. Myocardial infarction is the leading cause of death worldwide. Venous thromboembolism is also a common disease, with 1 to 2 people in 10 million suffering from acute venous thromboembolism each year [2]. Although anticoagulants can effectively treat patients with thrombosis, the risk of bleeding cannot be ignored. In addition, the formation of blood clots is closely related to platelets and coagulation function. Abnormal activation of platelets increases the risk of embolism and myocardial infarction [3]. A large number of studies have shown that mTOR is a key gene in regulating platelets and plays an important role in promoting platelet hyperreactivity and thrombosis [4, 5]. 

In traditional Chinese medicine (TCM), the treatment of thrombosis focuses on promoting blood circulation and removing blood stasis [6–8]. Among them, Leonurine is a ingredient of TCM for treating gynecological diseases, and has the effect of promoting blood circulation and regulating menstruation [9]. Leonurine is a natural small molecule compound derived from motherwort, which has low toxic side effects and shows great application potential in cardiovascular diseases [10]. For example, in atherosclerosis (AS), currently known therapeutic drugs such as statins have significant side effects on muscle while providing treatment [11]. Leonurine plays a role in AS by lowering cholesterol and has the potential to become an alternative to statins [9]. Li et al. find that Leonurine has anticoagulant and vasodilator effects in zebrafish, a model animal, and can reduce thrombosis by regulating platelet activation, amino acids, and inositol metabolites [12].

However, TCM is a complex system with multiple components, multiple targets and multiple pathways, which requires multi-level and multi-dimensional analysis and exploration. It is difficult to make breakthroughs by relying solely on traditional methods such as medicinal properties theory and clinical experience [13]. Interdisciplinary knowledge and methods, combined with experimental verification, can accelerate the research of TCM and achieve real and effective progress. Drug repositioning prediction is known for its low cost and high efficiency. In particular, with the improvement of the traditional Chinese medicine database, multimodal data such as known herbs, ingredients, genes, diseases, etc. have been scientifically summarized, which makes the application of repositioning models based on TCM data possible. Meanwhile, in order to discover the hidden components and disease information in complex multimodal networks, random walks have been widely used in bioinformatics applications due to their excellent interpretability. Bo-Wei Zhao et al. [14] calculate the topological features after performing random walks on the drug-protein-disease heterogeneous graph when predicting drug-disease relationships. Tong Zhang et al. [15] use a hypergraph random walk algorithm to calculate the importance scores of mutant genes and combined them with signaling pathway data to identify synergistic cancer driver genes in individual patients. Rhys Gillman et al. [12] use restarted random walks to identify subnetworks around each mutated gene in the network.

In this work, we use random walk and random forest methods to construct a Chinese medicine ingredients-diseases repositioning model, and combine network pharmacology analysis and biological experiments to study the potential diseases and mechanisms of Leonurine. Firstly, through two authoritative databases, the TCM Database ETCM [16] and the Protein Database STRING [17], we integrate multimodal data includes herbs, ingredients, diseases, genes, proteins and their interactions to construct a heterogeneous network. Then the random walk model is used to learn knowledge from the network and combined with the random forest model for relationship prediction. The diseases which related to Leonurine recommended by the model are sorted according to the prediction scores and combined network pharmacology experiments and wet experiments to verify from multiple aspects.

## Materials and methods

### Datasets

Multimodal data is collected from the Encyclopedia of Traditional Chinese Medicine (ETCM) [16], an encyclopedia of TCM. Entities include herbs, ingredients, genes, and diseases. Edges include ingredient-diseases, herbs-ingredients, ingredients-targets, herbs-targets, herbs-diseases, and diseases-targets. There are 402 herbs, 4289 ingredients, and 2661 diseases. In addition, the STRING database [17] is used to collect protein interaction networks. In this experiment, data with combined_score greater than or equal to 700 were screened from the STRING database to ensure that the interactions between the selected proteins have a high degree of credibility. A heterogeneous network ${\text{D}}_{\text{TCM}}$ is constructed by above data and shown in Table 1.

**Table 1:** The introduction of ${\text{D}}_{\text{TCM}}$

Relations	Node 1	Node 2	Edges	Source
Ingredients -Diseases	4029	2661	617530	ETCM
Herbs- Ingredients	402	6971	10304	ETCM
Herbs—Targets	398	1751	50061	ETCM
Diseases—Herbs	2704	395	248845	ETCM
Ingredients—Targets	4185	1635	75112	ETCM
Diseases—Targets	4289	4887	73540	ETCM
Targets—Targets	16201	16201	236943	STRING

### Ingredients -diseases repositioning model

**Fig. 1:**
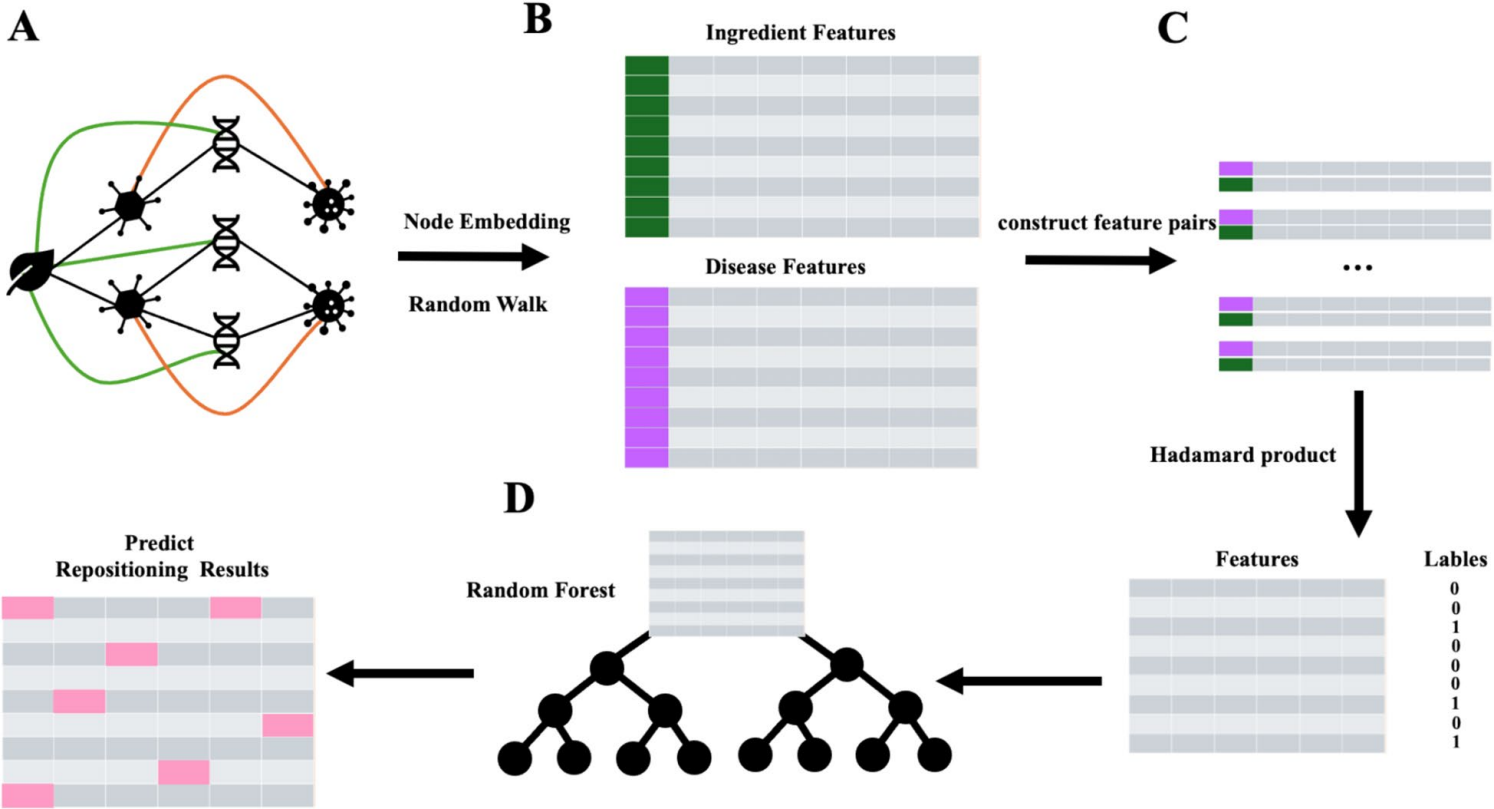
Ingredients-diseases relationship prediction model. **A**. Network construction. **B**. Network embedding representation. **C**. Ingredients-diseases similarity prediction. **D**. Predict repositioning results by random forest

The machine learning model used for the ingredient-disease repositioning task in this paper mainly consists of two modules, the node embedding module and the prediction module (Fig. 1). The heterogeneous network ${D}_{TCM}=(V,E)$ is used as the input of the model, and the node embedding module obtains the feature vectors of each component and disease as the input of the prediction module. The prediction module then uses the machine learning model to predict the relevant score of each ingredient -disease feature pair.

#### Node embedding module

The node embedding module is based on the random walk node2vec model [18]. It starts from a certain node and moves randomly between adjacent nodes according to specific probability rules to learn the embedded representation of components and diseases in the network. Node2vec combines the DFS (depth-first search) and BFS (breadth-first search) methods to enable adjustments during the walk to capture similarities at different levels and generate node sequences. Given the current node $v$ in the random walk model, the probability of visiting the next vertex $x$ is:

$$P\left({c}_{i}=x|{c}_{i-1}=v\right)=\left\{\begin{array}{c}\frac{{\pi }_{vx}}{Z} , if(v,x)\in E\\ 0 , otherwise\end{array}\right.$$

${\pi }_{vx}$ is the unnormalized transition probability between vertex $v$ and vertex $x$, $Z$ is a normalized constant, ${c}_{i}$ represents the $i$-th node in the random walk process, $(v,x)$ represents the edge between nodes $v$ and $x$, and $E$ represents the edge set. In general, the hyperparameters p and q control the random walk strategy [19]. When p is low, the walk will try to stay"locally"close to the starting node. The parameter q allows the search to distinguish between"local"and"global"nodes. If q > 1, the random walk has a greater probability of sampling nodes around the initial node. Assuming that the current random walk passes through the edge $(t,v)$ to reach vertex $v$, we get the coefficient ${\alpha }_{pq}\left(t,x\right)$ related to the current random walk state,

$${\alpha }_{pq}\left(t,x\right)=\left\{\begin{array}{c}\frac{1}{p}, if {d}_{tx}=0\\ 1, if {d}_{tx}=1 \\ \frac{1}{q}, if {d}_{tx}=2\end{array}\right.$$

where ${d}_{tx}$ is the shortest distance between vertex $t$ and vertex $x$.

And based on the current edge $\left(v,x\right)$, the next walk transition probability ${\pi }_{vx}$ is determined by,

$${\pi }_{vx}={\alpha }_{pq}(t,x)\bullet {w}_{vx}$$

where ${w}_{vx}$ is the edge weight between vertices $v$ and $x$.

**Fig. 2:**
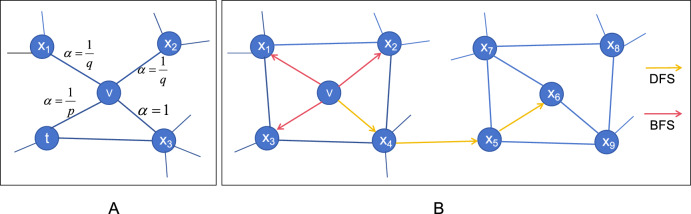
Diagram of random walk. **A**. Schematic diagram of node walk selection. **B**. Schematic diagram of BFS and DFS

The skip-gram model [20] is trained to learn node embeddings. The goal of the skip-gram model is to maximize the probability of the appearance of its neighbor node $u$ given a node $v$, as follows:

$$\mathit{max}\sum_{v\in V}\sum_{u\in N(u)}\mathit{log}P(u|v)$$

where $V$ is the node set, and $N(v)$ is the neighbor set of node $v$ (Fig. 2).

According to the node feature vector obtained by node2vec, we select node degree, node clustering coefficient, node proximity centrality and node PageRank value in combination with network topology attributes. Node degree refers to the number of edges directly connected to a node. Node clustering coefficient measures the closeness of interconnection between neighboring nodes of a node. Node proximity centrality reflects the average shortest path length from a node to all other nodes. Node PageRank value evaluates the nodes in the network structure. The direct splicing method is used to fuse the node embedding representation and network topology attributes. Finally, we get a component vector feature matrix of size 4028*68 and a disease vector feature matrix of size 2660*68.

#### Prediction module and evaluation metrics

In the prediction module (Fig. 1C), we pair the component features ${F}_{C}$ and disease features ${F}_{D}$ constructed by the node embedding module, and fuse the two into ${H}_{CD}$ using the Hadamard product. Then, the random forest (RF) model is selected as the classifier of the module [21]. By integrating multiple decision trees, RF can parallelize calculations and reduce the problem of overfitting of a single decision tree. Meanwhile, RF can handle high-dimensional data well through random feature selection and automatic feature selection. And by introducing randomness to reduce the correlation between individual decision trees, the stability and accuracy of the model are improved.

Finally, we use five evaluation indicators, AUC, AUPR, Accuracy, Precision, Recall, and F1-score, to evaluate the performance.

After completing the model training, we actually extract the natural ingredient Leonurine and its related diseases from the prediction results and sort them according to the scores to further analyze the potential therapeutic effects of Leonurine and thrombosis-related diseases.

### Network pharmacology analysis of relation between leonurine and thrombosis

First, the SwissTargetPrediction database [22] is used to collect potential targets of Leonurine. The Genecards database [23] is used to query target genes related to thrombosis. The STRING database is used to perform PPI network analysis on the screened common target genes to obtain the interaction network between these proteins. Subsequently, the Metascape database [24] is used to perform pathway enrichment analysis on the common target genes to identify the biological processes and signaling pathways in which these genes are mainly involved. Finally, network visualization software such as Cytoscape [25] is used, combined with pathway analysis results, to screen key target genes from the common target genes as candidate genes for subsequent experimental verification.

### SAB for calculating correlation between leonurine and thrombosis

**Fig. 3:**
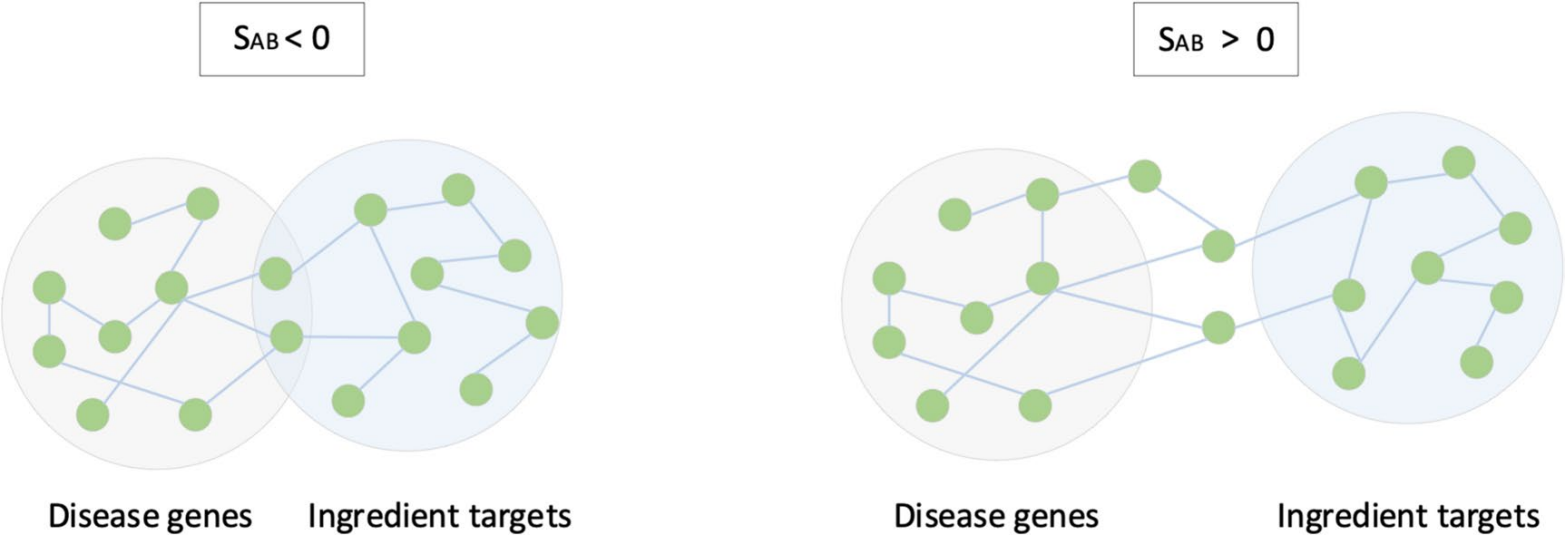
Diagram of SAB method

SAB is a method for measuring the correlation between two protein networks [26]. In this experiment, the correlation between Leonurine and thrombosis is evaluated by the SAB algorithm. As mentioned above, the ingredients and disease-corresponding target data are collected to construct the PPI network. In this method, the Leonurine gene set A and the thrombosis gene set B are regarded as two node sets, representing the gene groups involved in the protein interaction network. The correlation of the network is evaluated by comparing the protein shortest path distance (${d}_{AB}$) between Leonurine and thrombosis-related genes and the shortest path distance (${d}_{AA}$ and ${d}_{AB}$) of protein–protein pairs between groups. The specific calculation formula of SAB is shown as follows (Fig. 3).

$${S}_{AB}={d}_{AB}-\frac{{d}_{AA}+{d}_{AB}}{2}$$

### Molecular docking simulation

We collected mTOR protein structures (PDB: 2RSE) from PDB database [27] and performed molecular docking with Leonurine from PubChem dataset [28]. Select Discovery Studio molecular docking software for the platform. The protein structure is derived from the solution nuclear magnetic resonance of the lowest energy structure [29]. The processing flow includes: preparation of molecules in the default environment, setting of binding pocket, and docking through CDOCKER.

### Molecular dynamics

In order to determine the binding stability of mTOR and Leonurine, a 50 ns molecular dynamics simulation (MD) is used to evaluate the protein–ligand structure [30, 31]. Gromacs software is used to simulate the binding process [32]. First, the protein protonation state is processed at pH 7.4 in combination with pdb2pqr [33]. The ligand profile is generated using acpype [34]. The Amber force field and TIP3P water model are used to add the solution and 0.15 M Na + and CL- to a 1.2 nm cubic box. Subsequently, the stable equilibrium is carried out after energy minimization at a temperature of 300 K and a pressure of 1 bar. Finally, a 50 ns simulation is performed.

### Biological experiments

#### Cell experiments

1. Cell culture and model establishment: Select appropriate cell lines (human umbilical vein endothelial cell line HUVEC) for culture and establish thrombosis model cells. The model can be established by using tumor necrosis factor-α (TNF-α) as a stimulus to activate endothelial cells and induce tissue factor (TF) expression.
2. Drug treatment and concentration screening: The CCK-8 assay is employed to determine the optimal concentration of Leonurine for assessing its effect on endothelial cell proliferation. Additionally, Western Blotting is used to evaluate the effects of different concentrations of Leonurine on the expression of key proteins in endothelial cells.
3. Cell apoptosis detection: Flow cytometry is used to detect the apoptosis of thrombotic model cells after treatment with different concentrations of Leonurine. The apoptosis rates of normal endothelial cells, model cells, and cells treated with low and high concentrations of drugs are compared to evaluate the anti-apoptotic effect of Leonurine.
4. Western Blotting experiment: The total protein of the treated cells is extracted, and the expression level of mTOR protein is detected by Western Blotting technology. The specific steps included protein sample preparation, SDS-PAGE electrophoresis, membrane transfer, antibody incubation and color development. The expression changes of mTOR protein in normal endothelial cells, model cells and cells treated with different concentrations of Leonurine are compared and analyzed to verify the regulatory effect of Leonurine on the mTOR gene.

#### In vivo validation

1. Animal model verification: Thrombosis mouse model: Select appropriate strains of mice (such as C57BL/6 mice) and construct a thrombosis model through a specific surgical method (venous ligation). All animal experiments in this study adhere to relevant ethical guidelines and are approved by the corresponding ethics committee.
2. Control group and experimental group settings: The mice are randomly divided into two groups, one group as the control group, without any treatment; the other group as the Leonurine treatment group, given an appropriate dose of Leonurine for treatment.
3. Sample collection: At a specific time point (7 days after treatment), the mice are euthanized and the vascular tissue of the lesion is taken for subsequent experiments.
4. Tissue sectioning: the collected vascular tissue is fixed with 4% paraformaldehyde, dehydrated, transparent, and wax-impregnated to make a paraffin block. The paraffin block is cut into thin slices with a thickness of 4–5 microns using a slicer and attached to a glass slide.

Immunohistochemistry: Dewaxing and hydration: Place the sections in xylene and gradient ethanol for dewaxing and hydration treatment. Antigen retrieval: Use heat retrieval or enzyme retrieval methods to expose the antigens in the tissue. Blocking: Use serum or blocking solution to block the sections to reduce nonspecific staining.

Primary antibody incubation: Add diluted mTOR antibody, place in a humidified box, and incubate at 4 °C overnight or at room temperature for a certain period of time. Secondary antibody incubation: Use the secondary antibody corresponding to the primary antibody for incubation, usually at room temperature. Color development: Use DAB color development system or other appropriate color development methods to observe the expression of the target protein. Counterstaining and sealing: Use hematoxylin for counterstaining, dehydrate, and make transparent, and then seal with neutral gum.

- (5) Result observation and analysis: Use a microscope to observe the immunohistochemical sections, record the expression of mTOR protein in thrombus tissue, and compare and analyze with the control group. Image processing software can be further used for quantitative analysis, such as measuring indicators such as the number of positive cells and positive staining area.

## Results

### Performance of ingredients-diseases repositioning model

Based on heterogeneous networks, three random walk models and three classic machine learning classification models are used to compare to obtain the best prediction performance. The parameter settings of node2vec [18], Deepwalk [35], and mathpath2vec [36] are listed in Table 2. The parameter selection refers to existing work [37, 38]. In particular, the selection of p and q parameters of node2vec, due to the complexity of the TCM network, we prefer to assign feature information to the target node through closely related nodes. This is because of the characteristics of TCM network data, highly correlated disease-disease relationships or herb-disease relationships are more likely to show high correlation in the form of short distance and close links [39]. We randomly divided the dataset into training and test sets according to the 8:2 strategy. The results of the model test set are shown in Table 3, where the model combining node2vec and RF achieves the best prediction results. Especially the AUC indicator reaches 0.96, AUPR reaches 0.66. In addition, the Recall indicator reaches 0.9618. This indicates that despite severe data imbalance, the model has excellent ability to distinguish between positive and negative samples. Therefore, in the subsequent work, the node2vec joint RF model is selected as the prediction model.

**Table 2:** Random Forest Algorithms Parameters

Algorithms	Parameters
node2vec	dimensions:64,walk_length:50,num_walks:10, window:10,workers:4,p:0.5,q:2
Deepwalk	dimensions:64,walk_length:50,num_walks:10, window:10,workers:4
metapath2vec	dimensions:64,walk_length:50,num_walks:10, window:10,workers:4

**Table 3:** Performance of ingredients-diseases repositioning model

Methods	AUC	AUPR	Acc	Pre	Recall	F1
node2vec + RF	0.9618	0.6659	0.9735	0.8478	0.9618	0.8953
Deepwalk + RF	0.9507	0.6985	0.9781	0.8747	0.9507	0.9086
metapath2vec + RF	0.8242	0.2436	0.8866	0.6439	0.8242	0.6853

### Analysis of Leonurine-related diseases

After ensuring the validity of the model, we screened out diseases related to Leonurine from the model results (Table 4). In the model classification results, a total of 19 diseases are judged to be related to Leonurine. It is worth noting that in addition to the known atherosclerosis and bleeding diseases, a large number of thrombosis-related diseases also appears in the related diseases, including: Recurrent Thrombophlebitis, Thrombotic Disease, thrombosis. This shows that Leonurine have great application potential in thrombotic diseases.

**Table 4:** Leonurine-related diseases

Disease	True_Label	Predicted_Label
Recurrent thrombophlebitis	1	1
Thrombotic disease	1	1
Coagulative disorders	1	1
Coronary atherosclerosis	1	1
Prolonged whole-blood clotting time	0	1
Heparin-induced thrombocytopenia Type Ii	1	1
Ecchymosis	1	1
Abnormality of the respiratory system	1	1
Abnormality of the gallbladder	1	1
Gingival bleeding	1	1
Hereditary angioedema	1	1
Cerebral hemorrhage	0	1
Thrombosis	1	1
Ataxia	0	1
Recurrent deep vein thrombosis	0	1
Joint hemorrhage	1	1
Prolonged bleeding after dental extraction	0	1
Disseminated intravascular coagulation	0	1
Duodenal ulcer	1	1

The SwissTargetPrediction database is used to screen drug targets and identifies a total of 100 potential targets for Leonurine. 1777 target genes related to thrombosis are retrieved from the GeneCards database. Combined with the PPI network, an association network is constructed. At the same time, combined with SAB analysis, the correlation score between the two is − 0.3518. When the score is less than 0, it means that the component is closely associated with the disease.

### Theoretical verification results

**Fig. 4:**
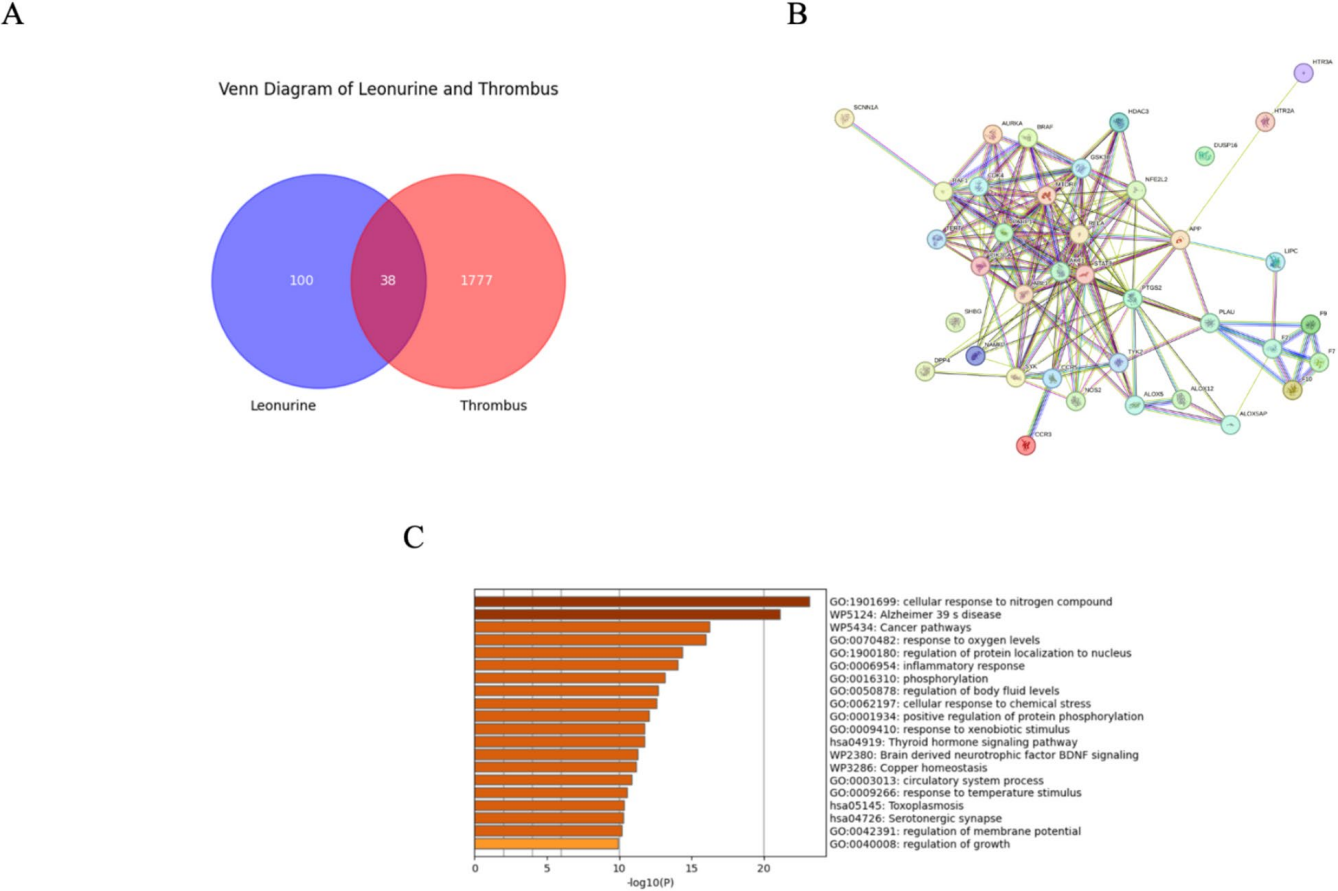
Network pharmacology analysis. **A**. VENN diagram of the intersection of Leonurine and thrombosis genes. **B**. Protein network interaction diagram. **C**. Bio-enrichment analysis

For the genes collected by SwissTargetPrediction and GeneCards databases, we also performed a complete bio-enrichment analysis. As shown in Fig. 4, the Venn diagram of Leonurine and potential targets of thrombosis shows that there are 38 intersection genes between the two. The PPI network analysis is performed on these 38 genes using the STRING database to construct an interaction network between proteins (Fig. 4B). In addition, the Metascape database is used for bio-enrichment analysis. The Cytoscape software is used to screen the top 5 target genes with the greatest influence from these 38 target genes, namely: STAT3, AKT1, PARP1, MTOR, and RELA.

As a core regulator of cell metabolism, mTOR mainly regulates metabolism and growth through the sensing of nutrients, energy, and oxygen, while other molecules such as STAT3 and AKT1 focus more on regulating cell proliferation, immune response, and repair processes. AKT1 and STAT3 are upstream signaling molecules that directly affect the activity of mTOR. PARP1 mainly interacts with the mTOR pathway in DNA repair and stress response, while RELA is involved in the regulation of immune and inflammatory responses, indirectly affecting cell metabolism [40, 41]. Studies have shown that PI3K/AKT1/mTOR plays a key role in anti-thrombosis. The activation of this pathway can inhibit platelet autophagy and promote platelet aggregation to form thrombi, while RELA, STAT3, and PARP1 indirectly regulate this pathway [42–44] (Fig. 4D).

**Fig. 5:**
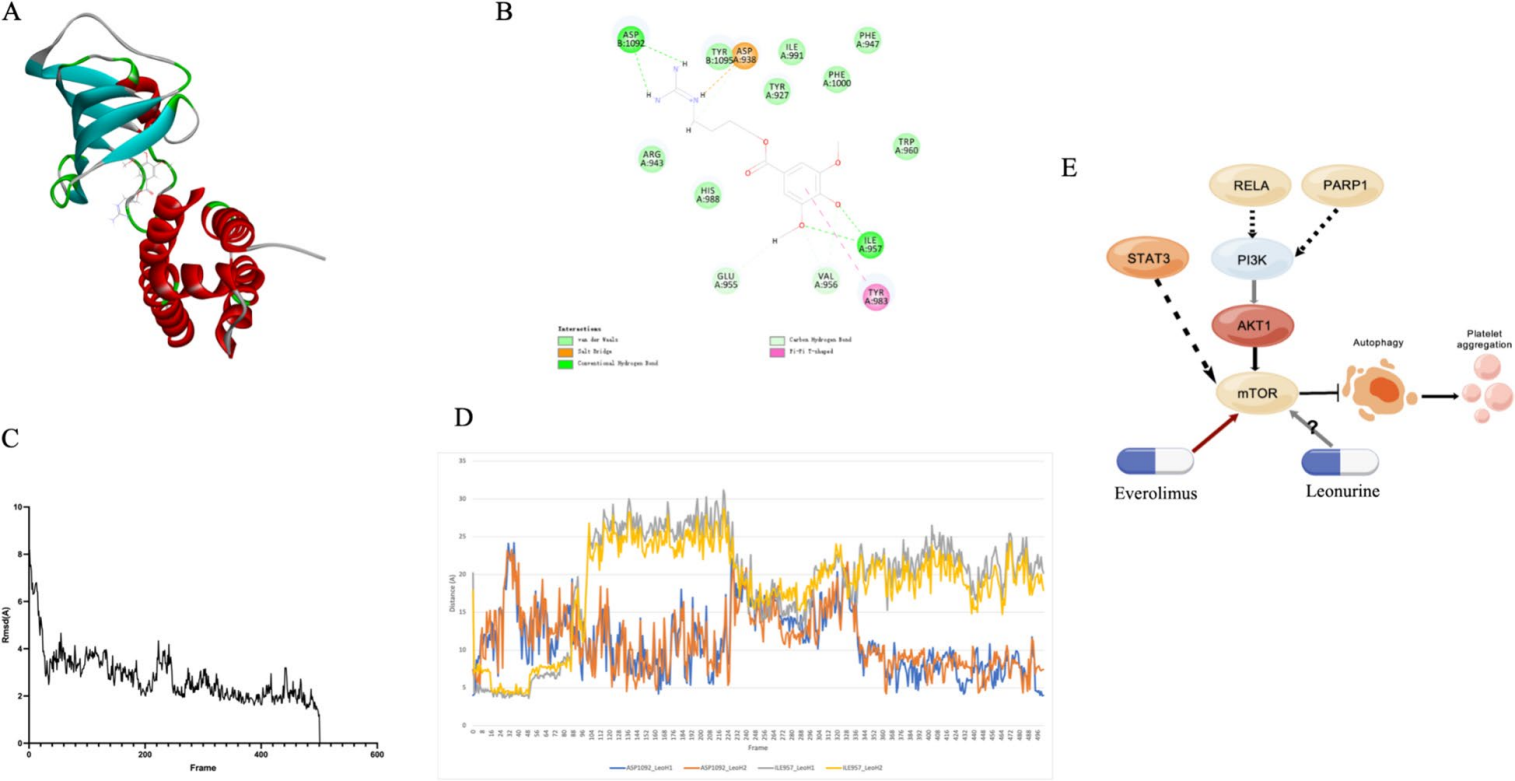
Molecular docking results and potential mechanism diagram. **A**. Leonurine is docked with mTOR protein (PDB: 2RSE). **B**. 2D docking bones diagram. **C**. The protein RMSD result of mTOR-Leonurine complex. **D**. shows the distance between ASP1092- Leonurine and ILE957- Leonurine during the MD. **E**. Potential mechanism of action. PI3K\AKT1\mTOR regulates platelet aggregation, and STAT3, RELA, and PARP1 have indirect effects on this pathway. Everolimus acts directly as a marketed drug, and Leonurine may also have a regulatory effect on mTOR

The mTOR protein structures (PDB:2RSE) we collected were complexes of mTOR and FKBP12 protein. Studies have shown that the FRB domain in mTOR forms a stable complex structure with the FKBP12 protein [29]. The pharmacological effects against mTOR are mainly explained and discussed in the context of forming complexes with the protein FKBP12. In the gap formed in the middle of the complex is the docking site of Rapamycin, which will specifically inhibit mTORC1 in mTOR [45]. We selected this docking site to dock with Leonurine, and the results are shown in Fig. 5A, B. In the optimal docking result, -CDOCKER_ENERGY is 31.8117 and -CDOCKER_INTERACTION_ENERGY is 43.9747. Among them, Leonurine will form a stable hydrogen bond with ASP1092 and ILE957 around the pocket, forming a salt bridge with ASP938.

In addition, we analyzed the binding stability of the mTOR-Leonurine complex by MD (see Additional file 1). In the generated 50 ns simulation results, a total of 502 frames are saved. When the simulation tends to be stable, the RMSD value of the protein is less than 3 $\dot{A}$ (Fig. 5C). At the same time, the molecular docking results screen out ASP1092 and ILE957 residues that form hydrogen bonds with Leonurine to help docking. Figure 5D shows the distance between the residues and Leonurine during MD. Especially after 350 frames, the distance change becomes stable, which is consistent with the RMSD trend.

Among the drugs circulating on the market, Everolimus, as an anti-tumor drug (DB01590), clearly acts on mTOR. Because of its effect and the mechanism of mTOR and inhibiting platelet aggregation, it has been used to study its potential role in anti-thrombosis and has published related research work in many internationally influential journals [46–48]. Among them, marketed Everolimus as a coating of platinum chromium alloy bracket system is a hot topic [49–51]. This system can not only help the rapid healing of the coronary artery, but also maintain an extremely low thrombosis rate. In particular, the pathways in which Everolimus is involved in proliferation, cell survival and angiogenesis are also the PI3K/AKT/mTOR pathway [52]. This provides sufficient confidence for the possibility of Leonurine in subsequent clinical trials and marketing applications.

Therefore, combined with multi-level theoretical analysis and verification, we believe that the mechanism diagram of Leonurine as a drug with great potential in cardiovascular disease. The mechanism diagram in antithrombotic applications may be shown in Fig. 5E, plays an important regulatory role through the mTOR site and has clinical value.

### Cell experiments

**Fig. 6:**
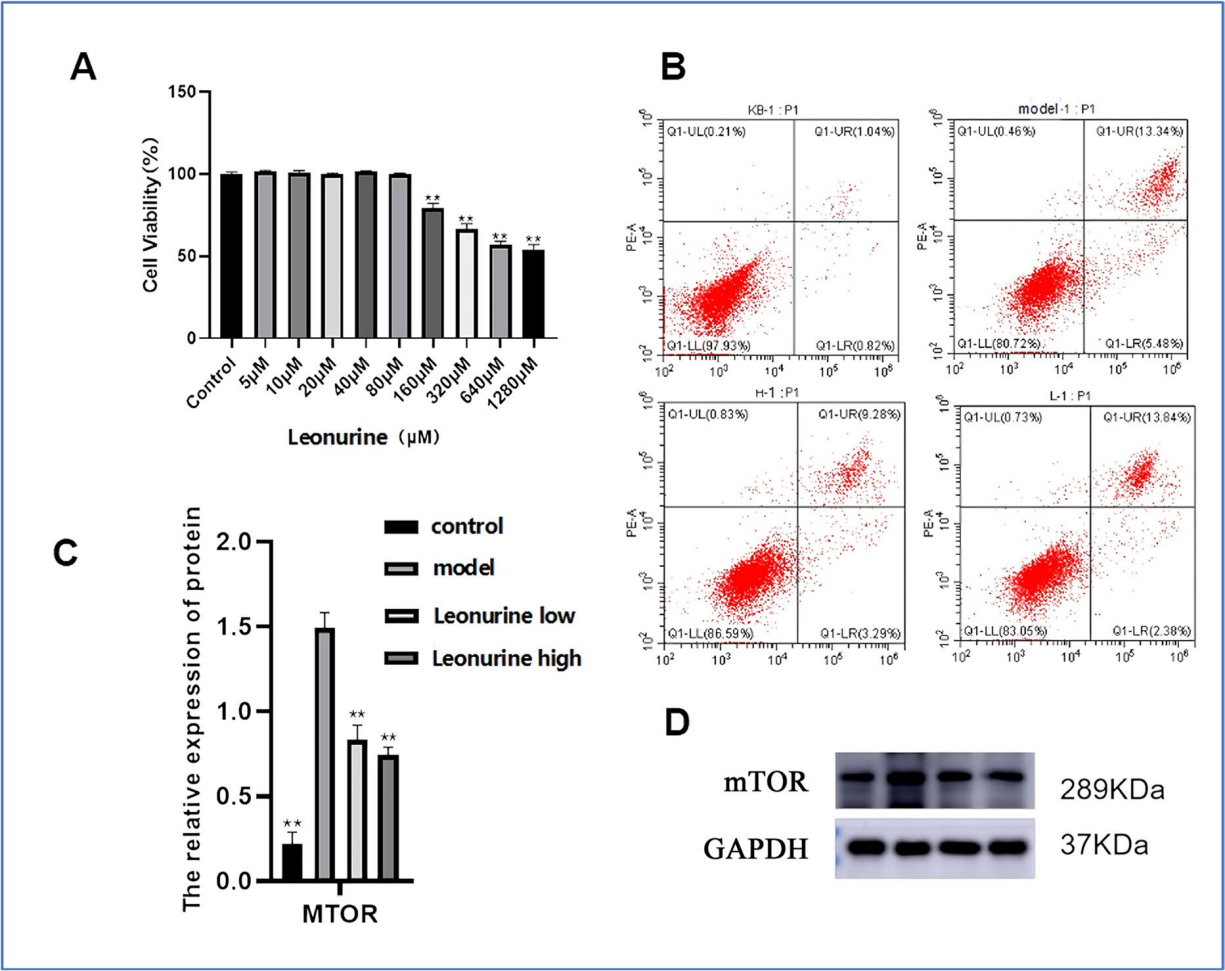
The relationship between mTOR gene and thrombosis. **A**. CCK-8 drug concentration screening. **B**. Cell apoptosis experiment. **C**. Western Blotting experiment. **D**. Protein electrophoresis. ** means p < 0.01

To further verify the relationship between the mTOR gene and thrombosis, we conducted cell and molecular experiments. First, Fig. 6A shows the effect of different concentrations of Leonurine on the activity of thrombotic cells. According to the results, 160 μM and 640 μM are used as low-concentration and high-concentration drug groups, respectively, for subsequent experimental verification. Figure 6B shows the apoptosis rate of thrombotic cells treated with different concentrations of Leonurine. The results show that the apoptosis rates of normal endothelial cells, thrombotic model cells, low-concentration Leonurine treatment group and high-concentration Leonurine treatment group are 1.86%, 18.82%, 16.22% and 12.57%, respectively.

In addition, Fig. 6C and D show the expression of mTOR protein after treatment with different concentrations of Leonurine. The results show that compared with normal endothelial cells, the expression of mTOR protein in thrombotic model cells is significantly increased. After treatment with Leonurine, the expression of mTOR protein decreases significantly, and the decrease in the high-concentration treatment group is more significant (P < 0.05). These results indicate that Leonurine can effectively regulate the expression of mTOR protein, supporting its potential therapeutic role in thrombosis.

### In vivo validation

**Fig. 7:**
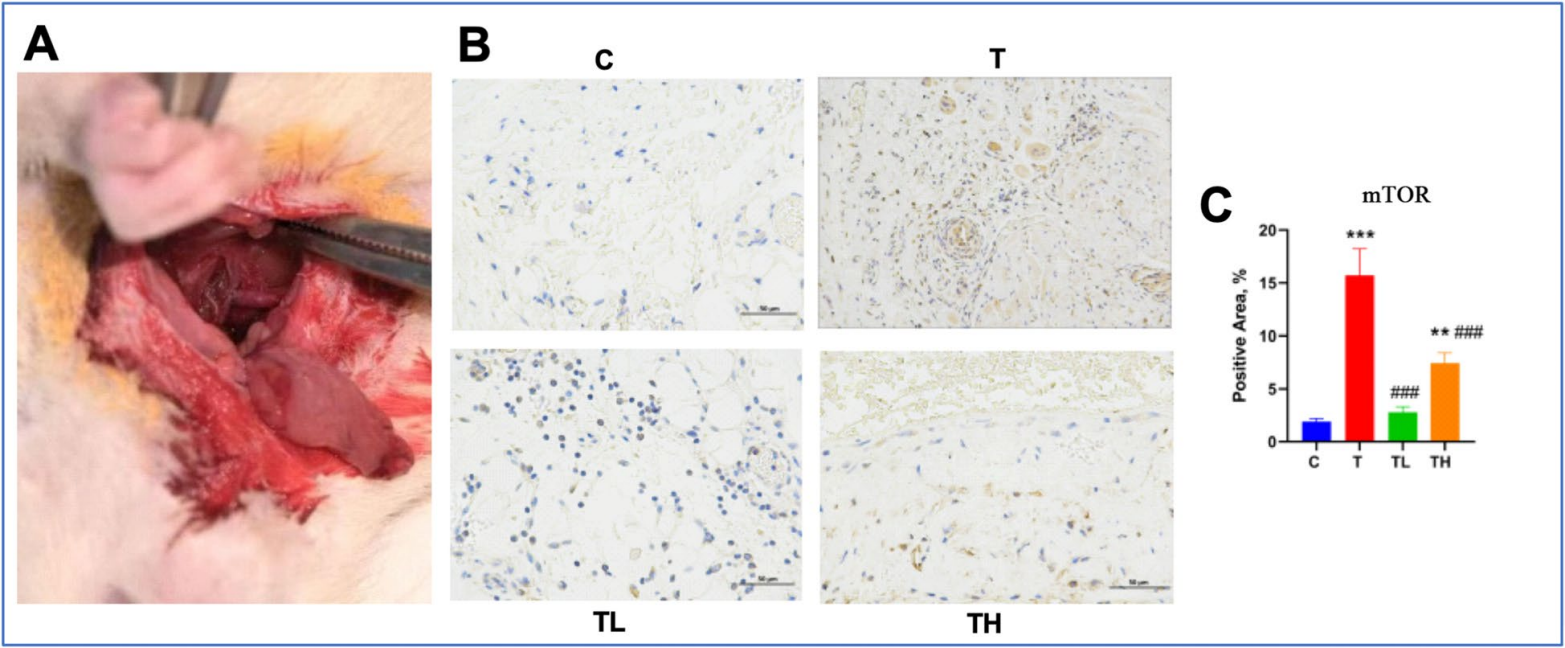
Effect of Leonurine on mTOR Gene Expression in Thrombotic Tissue. **A**. Venous Thromboembolism Mouse Model. **B**. Immunohistochemistry Images. B(c), Control Group. B(T), Model Groups. B(TL), The Model Group was treated with Low Concentration Drug. B(TH), The Model Group was treated with High Concentration Drug. **C**. Quantitative Analysis of Immunohistochemistry. ***, p < 0.001. **, p < 0.01. ###, p < 0.001. *, Compare with Control Group C. #, Compare with Model Group T

In order to further explore the regulatory effect of Leonurine on the expression of mTOR gene in the thrombotic mouse model, we conducted an immunohistochemistry experiment. The experimental results show (Fig. 7) that compared with the control group, the expression of mTOR in the model group without Leonurine is significantly increased (P < 0.05). Specifically, the expression level of mTOR protein is significantly decreased after Leonurine treatment, indicating that Leonurine may regulate the molecular mechanism related to thrombosis by downregulating the activity of the mTOR pathway.

The results show that Leonurine has a significant regulatory effect on the mTOR gene in the thrombotic mouse model, suggesting that it may exert its biological effects by regulating the mTOR signaling pathway. This finding provides an important basis for further understanding the mechanism of action of Leonurine in the treatment of thrombosis and lays the foundation for future related mechanism research.

## Discussion

With the advent of the big data era, the amount of data in the biomedical field has grown exponentially. Consequently, efficiently and accurately extracting valuable information from these vast datasets has become a critical challenge in contemporary research. To address this challenge, this study constructes a TCM ingredients-diseases relationship repositioning model based on the Encyclopedia of Traditional Chinese Medicine (ETCM) database. By integrating multi-source heterogeneous data, including herbs, ingredients, genes, proteins, and diseases, we established a complex heterogeneous network. This model, combined with the node2vec random walk algorithm and the RF classifier, effectively predicts potential associations between TCM ingredients and diseases.

Leonurine, the primary active ingredient of motherwort, has garnered significant attention due to its low toxicity and potential applications in cardiovascular diseases. In this study, the ingredient-disease repositioning model identified 19 diseases associated with Leonurine, of which thrombotic diseases constituted approximately 26%. This finding suggests that Leonurine may hold substantial promise for thrombosis treatment. Furthermore, using network pharmacology, we screened potential targets of Leonurine and identified thrombosis-related genes through the SwissTargetPrediction, GeneCards, and STRING databases. The SAB algorithm reveales a correlation score of −3.5 between Leonurine and thrombosis, indicating a strong biological connection.

Further analysis demonstrated that the mTOR gene plays a pivotal role in thrombosis development. Many studies have shown that mTOR, as a platelet regulator, affects thrombosis as a mediator in multiple signaling pathways [53–55]. This is consistent with the results we obtained through artificial intelligence-assisted screening, and also brings confidence to the application of computer science in the biomedical field. It is worth affirming that the treatment of thrombosis targeting mTOR has received widespread attention, including traditional stent systems coated with Everolimus or Zotarolimus [56] and new stents coated with metformin [57]. mTOR is not only involved in platelet activation but also regulates endothelial cell proliferation and angiogenesis. Through cell and mouse experiments, this study confirmes that Leonurine significantly reduces apoptosis in thrombotic cells, modulates the mTOR pathway, and promotes endothelial cell proliferation and angiogenesis.

Although this study has achieved notable results in predicting and analyzing the relationships between TCM ingredients and diseases, several areas warrant further exploration. First, while we have preliminarily validated the diseases associated with Leonurine, larger-scale clinical studies and additional experimental data are necessary to confirm its efficacy in treating thrombotic diseases. Second, thrombosis involves complex molecular mechanisms. Thus, beyond mTOR, other targets and pathways may also contribute to Leonurine's therapeutic effects. Consequently, future research should investigate the multi-target and multi-pathway mechanisms of Leonurine in thrombosis. Moreover, although the random walk and RF models used in this study are effective, reliance on existing databases and algorithms may lead to potential information gaps. Hence, future studies should focus on optimizing and validating the prediction model to enhance its accuracy and interpretability, thereby better guiding the clinical application of TCM.

## Conclusion

This study demonstrates the potential of Leonurine as a therapeutic agent for thrombosis through its regulation of the mTOR signaling pathway. By integrating multimodal data and employing machine learning algorithms, we have shown that Leonurine significantly impacts thrombosis by modulating platelet activation and promoting angiogenesis. Experimental validation through cell and mouse models further supports its anti-thrombotic effects. Nevertheless, future research is essential to fully elucidate Leonurine's clinical potential and multi-target mechanisms, thereby providing a more robust foundation for its application in modern medicine.

## Supplementary Information

Additional file 1.

## Data Availability

The code is available at https://github.com/dxxxin/RW_RF. Other data will be made available on request.

## Abbreviations

PPI: Protein–protein interaction
TCM: Traditional Chinese medicine
AS: Atherosclerosis
ETCM: Encyclopedia of Traditional Chinese Medicine
DFS: Depth-first search
BFS: Breadth-first search
RF: Random forest
TNF-α: Tumor necrosis factor-α
TF: Tissue factor
